# *Candida parapsilosis*: A systematic review to inform the World Health Organization fungal priority pathogens list

**DOI:** 10.1093/mmy/myad131

**Published:** 2024-06-27

**Authors:** Mrudhula Asogan, Hannah Yejin Kim, Sarah Kidd, Ana Alastruey-Izquierdo, Nelesh P Govender, Aiken Dao, Jong-Hee Shin, Jutta Heim, Nathan Paul Ford, Valeria Gigante, Hatim Sati, C Orla Morrissey, Jan-Willem Alffenaar, Justin Beardsley

**Affiliations:** Prince of Wales Hospital, South-Eastern Sydney LHD, Sydney, Australia; Sydney Institute of Infectious Diseases, University of Sydney, Sydney, New South Wales, Australia; Sydney Institute of Infectious Diseases, University of Sydney, Sydney, New South Wales, Australia; Sydney Pharmacy School, Faculty of Medicine and Health, University of Sydney, Sydney, New South Wales, Australia; Department of Pharmacy, Westmead Hospital, Westmead, New South Wales, Australia; National Mycology Reference Centre, SA Pathology, Adelaide, South Australia, Australia; Mycology Reference Laboratory, National Centre for Microbiology, Instituto de Salud Carlos III, Majadahonda, Madrid, Spain; National Institute for Communicable Diseases (Centre for Healthcare-Associated Infections, Antimicrobial Resistance and Mycoses), a Division of the National Health Laboratory Service and School of Pathology, Faculty of Health Sciences, University of the Witwatersrand, Johannesburg, South Africa; Division of Medical Microbiology, Faculty of Health Sciences, University of Cape Town, Cape Town, South Africa; Sydney Institute of Infectious Diseases, University of Sydney, Sydney, New South Wales, Australia; Westmead Institute for Medical Research and Children’s Hospital at Westmead, Western Sydney LHD, New South Wales, Australia; Westmead Hospital, Western Sydney LHD, Sydney, Australia; Department of Laboratory Medicine, Chonnam National University School of Medicine, Gwangju, South Korea; Helmholtz Association, Helmholtz Centre for Infection Research, Germany; Department of HIV, Viral Hepatitis and STIs, World Health Organization, Geneva, Switzerland; Centre for Infectious Disease Epidemiology and Research, School of Public Health and Family Medicine, University of Cape Town, Cape Town, South Africa; AMR Division, World Health Organization, Geneva, Switzerland; AMR Division, World Health Organization, Geneva, Switzerland; Department of Infectious Diseases, Alfred Health, Melbourne, Victoria, Australia; Monash University, Department of Infectious Diseases, Melbourne, Victoria, Australia; Sydney Institute of Infectious Diseases, University of Sydney, Sydney, New South Wales, Australia; Sydney Pharmacy School, Faculty of Medicine and Health, University of Sydney, Sydney, New South Wales, Australia; Westmead Hospital, Western Sydney LHD, Sydney, Australia; Sydney Institute of Infectious Diseases, University of Sydney, Sydney, New South Wales, Australia; Westmead Hospital, Western Sydney LHD, Sydney, Australia

**Keywords:** *Candida parapsilosis*, non-albicans *Candida*, immunosuppression, invasive candidiasis, fungal infection

## Abstract

*Candida parapsilosis* is globally distributed and recognised for causing an increasing proportion of invasive *Candida* infections. It is associated with high crude mortality in all age groups. It has been particularly associated with nosocomial outbreaks, particularly in association with the use of invasive medical devices such as central venous catheters. *Candida parapsilosis* is one of the pathogens considered in the WHO priority pathogens list, and this review was conducted to inform the ranking of the pathogen in the list. In this systematic review, we searched PubMed and Web of Science to find studies between 2011 and 2021 reporting on the following criteria for *C. parapsilosis* infections: mortality, morbidity (hospitalisation and disability), drug resistance, preventability, yearly incidence, and distribution/emergence. We identified 336 potentially relevant papers, of which 51 were included in the analyses. The included studies confirmed high mortality rates, ranging from 17.5% to 46.8%. Data on disability and sequelae were sparse. Many reports highlighted concerns with azole resistance, with resistance rates of >10% described in some regions. Annual incidence rates were relatively poorly described, although there was clear evidence that the proportion of candidaemia cases caused by *C. parapsilosis* increased over time. While this review summarises current data on *C.parapsilosis*, there remains an urgent need for ongoing research and surveillance to fully understand and manage this increasingly important pathogen.

## Introduction

*Candida* is a genus of yeasts that commonly inhabit the normal microbiome of both humans and animals. Many *Candida* species have the potential to cause invasive human infections and are associated with significant mortality, approaching 50% globally.^1^ Whilst *C. albicans* is historically the most common cause of candidaemia,^2^ non-albicans*Candida* (NAC) species are becoming increasingly prominent as causative organisms and are of public health concern.

*Candida parapsilosis* is amongst these clinically significant NAC species, especially in nosocomial settings. *Candida parapsilosis* is currently the second or third most isolated *Candida* species in intensive care unit (ICU) populations globally.^3,4^ Other at-risk groups include those receiving total parenteral nutrition and critically ill neonates,^5^ partly due to the propensity of *C. parapsilosis sensu stricto* (and closely related cryptic species *C. metapsilosis* and *C. orthopsilosis*) to form biofilm on indwelling central venous catheters (CVC). However, horizontal transmissions from healthcare workers to inpatients are an added concern in *C. parapsilosis* infection outbreaks.^5^ Overall, *C. parapsilosis* is associated with lower mortality than *C. albicans*, likely related to its lower virulence. Nevertheless, the mortality rate of *C. parapsilosis* remains unacceptably high.^5^ Another concern with *C. parapsilosis* is that resistance to azole and echinocandin antifungal agents appears to be rising.^5^

Despite global concerns about *C. parapsilosis*, there are limited data on rates of antifungal resistance, population-level morbidity, its impact on healthcare systems, and prevention and control strategies. This systematic review aimed to comprehensively assess several critical aspects of *C. parapsilosis* infection. We summarised data published between 2011 and 2022 on features including mortality, morbidity, drug resistance, preventability, incidence rates, and geographic distribution patterns to inform the World Health Organizations Fungal Priority Pathogen List (WHO FPPL). We highlight research gaps to inform public health priorities, guide future research, and advocate for improved surveillance.

## Methods

After conducting pilot searches to optimise the balance between comprehensiveness and feasibility, we searched the PubMed and Web of Science databases from 1 January 2011 to 18 February 2021. For PubMed, the search was optimised using medical subject headings (MeSH) and/or keyword terms in the title/abstract for each pathogen and criterion. The final search used (*Candida parapsilosis* [MeSH terms]) combined, using AND term, with criteria terms including (mortality [MeSH terms]) OR (morbidity [MeSH Terms]) OR (hospitalisation [MeSH terms]) OR (disability [all fields]) OR (drug resistance, fungal [MeSH terms]) OR (prevention and control [MeSH subheading]) OR (disease transmission, infectious [MeSH terms]) OR (diagnostic [title/abstract]) OR (antifungal agents [MeSH terms]) OR (epidemiology [MeSH terms]) OR (surveillance [title/abstract]).

On Web of Science, MeSH terms are not available, so topic search (TS), title (TI), or abstract (AB) search were used. The final search used (TI = [‘*Candida parapsilosis*’] OR TI = [‘*C. parapsilosis*’]), combined, using AND term, with criteria terms each as topic search, including (mortality) OR (case fatality) OR (morbidity) OR (hospitali*ation) OR (disability) OR (drug resistance) OR (prevention and control) OR (disease transmission) OR (diagnostic) OR (antifungal agents) OR (epidemiology) OR (surveillance). Symbol * allows a truncation search for variations of the term (e.g., hospitalisation or hospitalization). Of note, the search was not limited to invasive human infections caused by *C. parapsilosis*.

Eligible articles were those reporting data on *C. parapsilosis* relevant to at least one criterion. Studies could be retrospective or prospective observational studies, randomised controlled trials, or clinical/laboratory surveillance reports. Excluded articles included those reporting on non-human data (e.g., animals, plants), case reports/conference abstracts/reviews, novel drug studies (preclinical, early phase, or non-randomised controlled trials), *in vitro* papers on resistance mechanisms, and papers not published in English.

The search results from each database were incorporated into Covidence® (Veritas Health Innovation, Australia), and duplicates were removed. Two researchers (H.Y.K. and M.A.) independently screened titles and abstracts in parallel based on the inclusion criteria, although no reason was recorded for exclusion during this phase. The remaining articles were subjected to full-text screening for inclusion by the same two researchers. Reasons for exclusion were recorded for all articles during this phase. In both phases, discrepancies were resolved by a third reviewer (J.W.A.). We added any relevant articles identified from full-text screening.

We extracted data from all remaining articles for relevant criteria using standardised report forms within Covidence® (H.Y.K. and M.A.). The extracted data were checked by the second reviewer (M.A.).

We conducted risk of bias assessments for all included studies using the most suitable tools available: the risk of bias tool for randomised trials version 2 (ROB 2) for randomised controlled trials^6^ and the risk of bias in non-randomised studies (RoBANS) tool for non-randomised studies.^7^ We used each criterion as an outcome of the study and assessed if any bias was expected based on the study design, data collection, or analysis in that study. Studies classified as unclear or high overall risk were still considered for analysis, although the risk of bias was highlighted in analyses.

## Results

**Figure 1.**
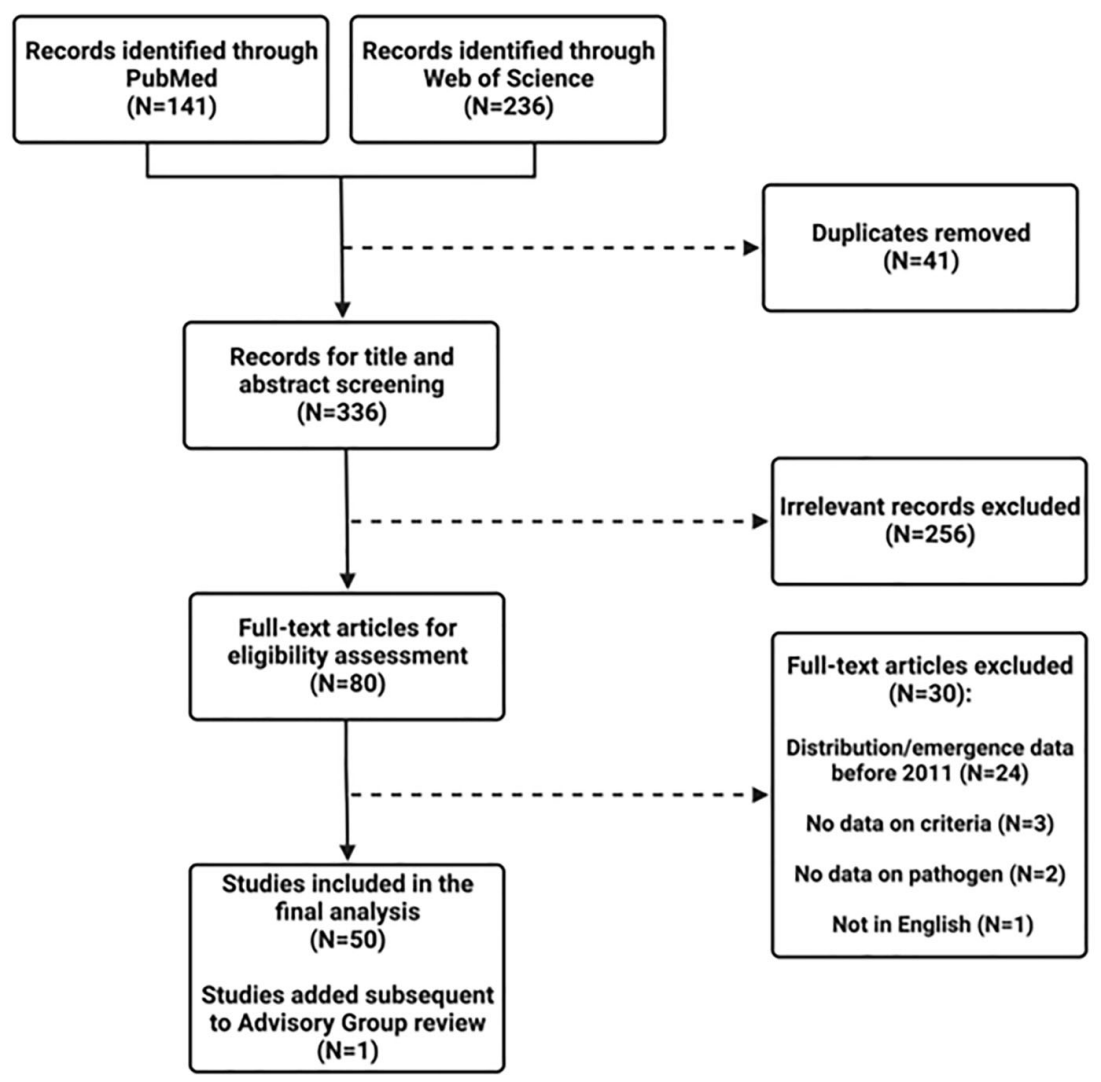
Flow diagram for selection of studies included in the systematic review.

PubMed and Web of Science Core Collection databases yielded 141 and 236 articles, respectively. After we removed duplicates, 336 articles remained for title/abstract screening. After excluding non-relevant articles, 80 underwent full-text screening, and 51 studies were included in the final analysis. A flow diagram outlining the process of study selection is shown in Figure 1.

The risk of bias for each study was assessed, and it is presented in Table 1. Of the included studies, 23 were classified as having a low risk of bias in all domains assessed. A total of 20 studies were classified as unclear risk of bias, mostly due to the selection biases caused by unclear eligibility criteria or population groups or unclear confirmation/consideration of confounding variables. Eight studies were classified as high risk because of selective reporting of results and non-consideration of confounding variables. A summary of the risk assessments is provided to offer transparency and context for the findings.

**Table 1.** Risk of bias.

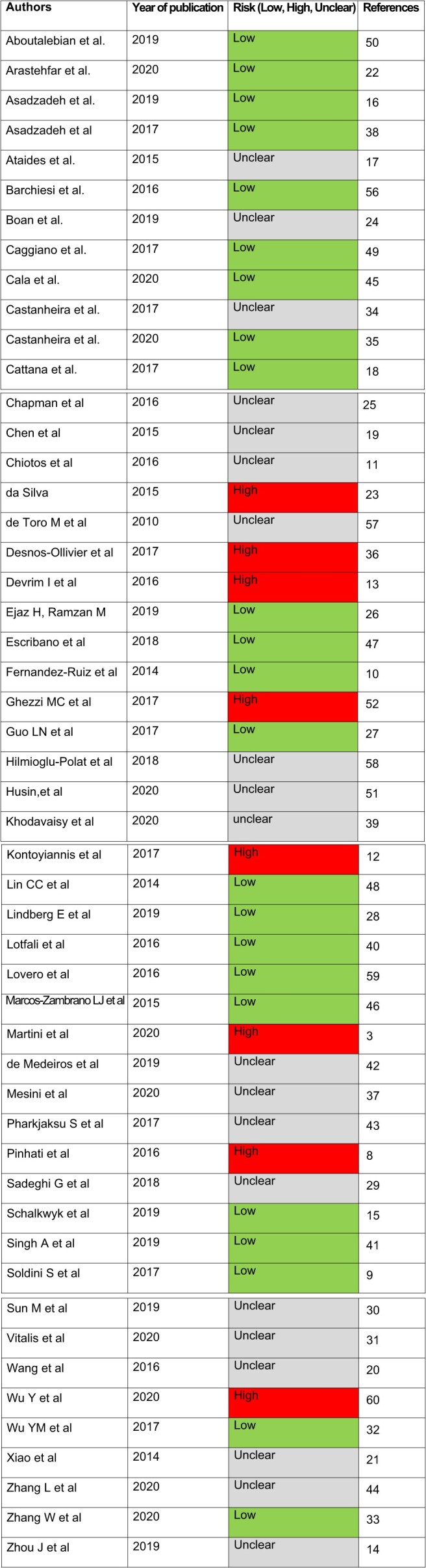

### Deaths

In total, 17 studies reported mortality for invasive infections caused by *C. parapsilosis*. Using heterogeneous time points encompassing early and late mortality, the crude rates ranged from 14.5% to 47% (Table 2 ). The studies ranged in size from 40 to 660 patients and were mostly retrospective. A total of 13 studies reported 30-day mortality: two studies, from Brazil^8^ and Italy,^9^ reported high 30-day mortality rates of 45% (18/40) and 46.8% (89/190), respectively, whilst the remaining studies with 30-day mortality reported rates of 17.5%–32.4%. One large study performed a sub-analysis specific for *C. parapsilosis* from a previous prospective trial in Spain^10^ and reported a 30-day all-cause mortality rate of 24.2%. In contrast, a lower 30-day mortality rate was reported (9.8%) in the USA in 2016.^11^ One study reported a 14-day mortality rate of 5.7% for a pooled cohort of unclear origin, although the study had a high risk of bias due to restrictive inclusion criteria.^12^

**Table 2.** Mortality.

Author, year	Study design	Study design	Study period	Country	Level of care	Population description	Number of patients	Mortality type	Mortality rate (*n/N*, %)
Arastehfar, 2020	Retrospective cohort study	Multi-centre	2015–2019	Iran	Tertiary	*Candida parapsilosis* complex candidaemia	90	In-hospital mortality	42/90 (46.6%)
Barchiesi, 2016	Retrospective cohort study	Single-centre	January 2010–December 2014	Italy	Tertiary	Patients with candidaemia	63	30-day mortality	Mortality 30 day: 17/63 (27% for *C. parapsilosis* and 53.9% for *C. albicans*) Early mortality (days 1–7): 4 (6%) Late mortality (days 8–30): 13 (21%)
Chen, 2015	Retrospective cohort study	Single-centre	2000–2012	Taiwan	Tertiary	Adults >18 with candidaemia	323	30-day crude mortality	80/323 (25%)
Chiotos, 2016	Retrospective cohort study	Multi-centre	January 2009–December 2013	USA	Adult inpatients (mixed levels of care)	Adults >17 years with candidaemia, who, survived for at least 4 days after the positive culture was obtained, and received initial therapy with only fluconazole or only an echinocandin	307	30-day crude mortality	9.8% 9.9% echinocandin group 9.5% fluconazole group (OR 1.05, 95% CI 0.49–2.26)
Devrim, 2016	Retrospective cohort study	Single-centre	December 2011–December 2013	Turkey	Tertiary paediatric intensive care	Paediatric patients with candidaemia treated with caspofungin	53	In-hospital and 30-day mortality	In-hospital: 20/53 (37.75%) 30-day mortality 4/53 (7.5%)
Fernandez-Ruiz, 2014	Other: subanalysis of completed prospective, multi-centre, population-based surveillance programme		May 2010–April 2011	Spain	Mixed	Mixed adult, paediatric and neonatal with candidaemia	190	30-day all cause	45/186 (24.20%)
Kontoyiannis, 2017	Other: pooled analysis—Pfizer study	Multi-centre	Not stated	Not stated	Not stated	Adult ≥18 with candidaemia	70	All-cause mortality	4/70 (5.7%) at 14 days 10/70 (14.3%) at 28 days
Lin et al., 2015	Retrospective cohort study	Single-centre	June 2008–June 2012	Taiwan	Tertiary	Mixed adult and paediatric patients with candidaemia	77	90-day mortality rate	90-day mortality: 32/77 (41.6%)
Medeiros, 2019	Retrospective cohort study	Single-centre	January 2011–December 2016	Brazil	Tertiary	Adult (+1 child) with candidaemia	68 (21.6% *C. parapsilosis*)	7-day mortality and 30-day mortality	30-day mortality: 27.3% for *C. parapsilosis* 61.1% for *C. albicans* 7 day all candidaemia: 23/68 (33.8%) 30 day all candidaemia: 38/68 (55.9%)
Mesini, 2020	Other: Prospective cohort with retrospective cohort inclusion	Single-centre	2008–20112012–2016	Italy	Tertiary	Adults with candidaemia	660	Mortality at 30 days	32.40% for *C. parapsilosis* 49.2% for *C. albicans*
Pinhati, 2016	Cross sectional study	Single-centre	July 2011–February 2012	Brazil	Tertiary	Adults ≥18 with candidaemia	40	Crude mortality at 30 days	18/40 (45%) for all *C. parapsilosis* 42.9% for fluconazole resistant *C. parapsilosis* 47.4% for fluconazole susceptible *C. parapsilosis*
van Schalkwyk, 2019	Lab surveillance	Multi-centre	2016–2017	South Africa	Various	All patients with candidaemia	2600 (516 with clinical outcome data)	In-hospital case fatality	166/516 (32.2%)
Soldini, 2018	Retrospective cohort study	Single-centre	2005–2015	Italy	Tertiary	Adults with candidaemia	190	7 days 30 days	40/190 (21%) 89/190 (46.8%)
Sun, 2019	Retrospective cohort study	Single-centre	1 March 2012–28 February 2018	China	Tertiary	Adults and paediatric	All *Candida* species 323*C. parapsilosis* 120	30-day mortality	14.6% for all *Candida* spp. 17.5% for *C. parapsilosis*
Vitalis, 2020	Retrospective cohort study	Single-centre	January 2013–December 2018	Hungary	Tertiary	Patients with candidaemia	127	30-day mortality	30%
Wu, 2018	Retrospective cohort study	Single-centre	1 January 2010–31 December 2010	Taiwan	Tertiary and primary	Hospitalised patients >18 years old with candidaemia	All *Candida* species 253 *C. parapsilosis* 60	30-day mortality	21.70% for *C. parapsilosis* 53.9% for *C. albicans*
Zhou, 2019	Retrospective cohort study	Single-centre	January 2012–December 2017	China	Tertiary	Major burn injury complicated by candidaemia	410	Attributed mortality	14.99% for all candidaemia (95% CI 10.24%–19.74%) Note: *C. parapsilosis* was most common species, but not the only species. No *C. parapsilosis* specific data

**Table 3.** Length of stay.

Author	Publication year	Study type	Study design	Study period	Country	Level of care	Fungal pathogen	Population description	Number of patients	Length of stay
Chiotos et al.	2016	Retrospective cohort study	Multi-centre	January 2009–December 2013	USA	Adult inpatients	*Candida parapsilosis*	Patients >17 years old	307	18 days
Devrim et al.	2016	Retrospective cohort study	Single-centre	December 2011–December 2013	Turkey	Tertiary paediatric intensive care	*Candida parapsilosis* complex	Paediatric	53	MIC <2 median 65 days (26–277) MIC = 2 median 67 days (15–484)
Soldini et al.	2017	Retrospective cohort study	Single-centre	2005–2015	Italy	Tertiary	*Candida parapsilosis sensu stricto*	Adult	190	40 days (IQR 26–62)
van Schalkwyk	2019	Lab surveillance study	Multi-centre	2016–2017	South Africa	Various	*Candida parapsilosis*	All patients with candidaemia	2600 (561 with outcome data)	40 days (IQR 25–59)

We found that several mortality studies had a high risk of bias due to restrictive inclusion criteria. The study from the USA reporting a mortality rate of 9.8% included only inpatients who survived for at least 4 days after the positive culture was obtained and received initial therapy with only fluconazole or an echinocandin. A study from Turkey included only patients treated with caspofungin and reported a case fatality of 37.8% and a 30-day mortality of 7.5%.^13^ Another study pooled data from six prospective cohorts but excluded patients from studies if they had received >48 h of prior antifungal therapy, had prosthetic devices at infection sites that were not able to be removed within 24 h of study entry, or had previously failed treatment for the current episode of *Candida* infection; this study reported mortality of 14.3% by 28 days.^12^ Finally, a retrospective study from China included all patients with candidaemia, although *C. parapsilosis* was the major cause, and reported a mortality rate of 15%.^14^

### Inpatient care

Four studies reported the length of hospital stays for *C. parapsilosis* infection,^9,11,13,15^ with medians ranging from 18 to 67 days. One study of paediatric patients reported a significantly longer length of stay for isolates with caspofungin minimum inhibitory concentration (MIC) of >2 µg/ml vs. ≤2 µg/ml (median 67 days, range 14–484 days vs. 65 days, range 26–277).^13^ The median lengths of stay reported among adults from three separate studies were 18 and 40 days.^9,11,15^ However, given the heterogeneity of patients and the small number of studies reporting this metric, no comment can be made on the length of stay directly attributable to *C. parapsilosis* infection.

### Complications and sequelae

No studies reported complications or sequelae specifically related to *C. parapsilosis* infection.

### Antifungal resistance

There were 43 studies on antifungal susceptibility, and most of these studies focused on blood isolates. We have reported the susceptibility of non-sterile site isolates since these may reflect colonising organisms causing invasive infections. An overview of the study type, location, source, and number of isolates is presented in Table 4. Six studies specifically reported MICs for *C. parapsilosis sensu stricto*.^16–21^ Whilst five studies reported identifying other members of the *C. parapsilosis* species-complex (*C. orthopsilosis* and *C. metapsilosis*),^10,18,19,22,23^ two studies did not provide specific susceptibilities for *C. metapsilosis* and *C. orthopsilosis* due to low isolate numbers.^18,22^ The predominant study type was retrospective cohort (*n* = 26), followed by laboratory surveillance studies either retrospectively or prospectively collected (*n* = 11). The remaining studies comprised three prospective cohort studies: one *in vitro* laboratory study, one cross-sectional study, and one pooled study sponsored by a pharmaceutical company. The study size ranged from 39 to 1153 isolates, with many studying multiple *Candida* species and providing some data specific to *C. parapsilosis* (which accounted for between 10% and 43% of all isolates; see below in the section on distribution).^8,14,20,21,24–33^ Most studies were performed in high-income or upper-middle-income countries (World Bank classification), with no studies in low-income countries; there was only one study from an African nation during the study window.

**Table 4.** Studies reporting drug susceptibility.

Author, year	Study type	Study design	Study period	Country	Level of care	Population description	Number of isolates	Samples collected from
Arastehfar, 2020	Retrospective cohort study	Multi-centre	2015–2019	Iran	Tertiary	Patients with *C. parapsilosis* complex	98 94/98 *C. parapsilosis* 4/98 *C. orthopsilosis*	Blood
Asadzadeh, 2019	Retrospective cohort study	Multi-centre	January 2010–August 2014	Kuwait	Tertiary	NICU patients with candidaemia due to *C. parapsilosis*	88 *C. parapsilosis sensu stricto* (72 neonates, 16 adults)	Blood (majority)
Asadzadeh, 2017	Retrospective cohort study	Multi-centre	January 2012–December 2015	Kuwait	Mixed	Mixed adult and paediatric	442	Blood (175) Urine (43) Sputum (24) Ear swab (29) Endotracheal aspirate (22) Line tip (9) Wound swab (6) Umbilical swab (4) Other (23)
Ataides, 2015	Retrospective cohort study	Single-centre	2007–2012	Brazil	Tertiary	Adult patients with candidaemia (*n* = 54) and onychomycosis (*n* = 33)	87 *C. parapsilosis* complex 78/87 *C. parapsilosis sensu stricto*, 5/87 *C. orthopsilosis*, 4/87 *C. metapsilosis*	Blood Nail
Barchiesi, 2016	Retrospective cohort study	Single-centre	January 2010–December 2014	Italy	Tertiary	Patients with candidaemia	63 *Candida parapsilosis* complex	Blood
Boan, 2019	Retrospective cohort study	Multi-centre	January 2005–December 2014	Australia	Tertiary	Adult patients with candidaemia	173 *Candida* spp. 25/173 *C. parapsilosis*	Blood
Caggiano, 2017	Retrospective cohort study	Single-centre	January 2007–December 2015	Italy	Tertiary	NICU patients with candidaemia	41 total 25/41 *C. parapsilosis* complex	Blood
Castanheira, 2017	Retrospective cohort study	Multi-centre	2014–2015	Global collection	Not stated	Invasive yeast and moulds collected in 29 countries worldwide	417 *C. parapsilosis*	Not stated
Castanheira, 2020	Retrospective cohort study	Multi-centre	January 2016–December 2017	25 countries	Tertiary	Patients from 60 hospitals	431 *C. parapsilosis*	Not stated
Cattana, 2017	Retrospective cohort study	Multi-centre	2009–2014	Argentina	Tertiary	Neonates and paediatric patients with invasive fungal infections	59/60 *C. parapsilosis sensu stricto* 1/60 *C. orthopsilosis*	Blood (29/60) Catheter (31/60)
Chapman, 2016	Prospective cohort study	Multi-centre	2014–2015	Australia	Tertiary	Patients with candidaemia	498/548 total *Candida* spp. with confirmed identification and AST 82/498 *C. parapsilsosis*	Blood
Chen, 2015	Retrospective cohort study	Single-centre	2000–2012	Taiwan	Tertiary	Adults (>18)	323 (256/323 *C. parapsilosis sensu stricto*, 33/323 *C. metapsilosis*, 34/323 *C. orthopsilosis*)	Blood
Da Silva, 2015	Other: laboratory-based surveillance/*in vitro* study	Multi-centre	08/2011–08/2013	Brazil	Mixed	Not described	81	Blood Urine Catheter tip Secretions Nails Skin
Desnos-Ollivier, 2018	Retrospective cohort study	Multi-centre	2003–2013	Uruguay, France (and Caribbean islands)	Mixed	Not described	161	Blood (majority) Ewe’s milk (*N* = 1)
Devrim, 2016	Retrospective cohort study	Single-centre	December 2011–December 2013	Turkey	Tertiary paediatric intensive care	Paediatric	53	Blood
Ejaz, 2019	Prospective cohort study	Single-centre	June 2015–May 2016	Pakistan	Tertiary	Paediatric	87 all *Candida* spp. 31/87 (35.63%) *C. parapsilosis*	Blood (68) Urine (19)
Fernandez-Ruiz, 2014	Other: sub-analysis of previous completed prospective, multi-centre, population-based surveillance programme	Multi-centre	May 2010–April 2011	Spain	Mixed	Mixed adult, paediatric and neonatal	194	Blood
Ghezzi, 2017	Retrospective cohort study	Single- centre	January 2010–December 2015	Italy	Tertiary	Mixed adult, paediatric and neonatal	514 *Candida* spp. 189/514 *C. parapsilosis* complex	Blood and intravascular devices
Guo, 2017	Other: prospective surveillance study	Multi-centre	January 2012–December 2013	China	Tertiary	Mixed adult and paediatric	1153 *Candida* spp. 161/1153 *C. parapsilosis* complex	Blood plus other ‘normally sterile sites’
Hilmioglu-Polat, 2018	Other: laboratory antifungal susceptibility study	Single- centre	2006–2014	Turkey	Tertiary	Not described	170	Blood
Khodavaisy, 2020	Other: laboratory antifungal susceptibility study	Multi-centre	March 2015–November 2018	Iran	Not stated	Adult and paediatric	101	Mixed = mostly nail (60%) Blood 15.8% Others
Kontoyiannis, 2017	Other: pooled analysis—Pfizer study	Multi-centre	Not stated	Not stated	Not stated	Adult ≥18	58 available with susceptibility testing	Blood Others: reported susceptibility data included non-blood isolates
Lin, 2015	Retrospective cohort study	Single-centre	June 2008–June 2012	Taiwan	Tertiary	Mixed adult and paediatric	77	Blood
Lindberg, 2019	Retrospective cohort study	Single-centre	January 2013–June 2016	Sweden	Mixed microbiology laboratory at university hospital. Samples received from regional centres included	Mixed adult and paediatric	233 *Candida* spp. 29/233 *C. parapsilosis*	Blood
Lotfali, 2016	Other: laboratory susceptibility study	Multi-centre	2009–2013	Iran	Not stated	Adult and paediatric	120	Mixed (non-blood)
Lovero, 2016	Other: prospective laboratory susceptibility study	Single-centre	January 2007–December 2014	Italy	University Hospital	Mixed adult and paediatric	163	Blood
Martini, 2020	Other: retrospective laboratory surveillance study	Single-centre	May 2014–May 2019	Italy	Tertiary	Mixed adult and paediatric	241	Blood
Medeiros, 2019	Retrospective cohort study	Single-centre	January 2011–December 2016	Brazil	Tertiary	Adult (+1 child)	51	Blood
Mesini, 2020	Other: prospective cohort with retrospective cohort inclusion	Single-centre	2008–2011 2012–2016	Italy	Tertiary	Adult	694	Blood
Pharkjaksu, 2018	Other: laboratory epidemiology and sensitivity study	Single-centre	January 2011–December 2015	Thailand	Tertiary	Not described	139	Blood and sterile sites
Pinhati, 2016	Cross sectional study	Single-centre	July 2011–February 2012	Brazil	Tertiary	Adults >/= 18	40 *Candida* spp. 28/40 *C. parapsilosis*	Blood
Sadeghi, 2018	Prospective cohort study	Single-centre	2014	Iran	Not reported	Mixed adult and paediatric	79 non-Candida *albicans* 29/79 *C. parapsilosis*	Skin Nail Vulvovaginal
Singh, 2019	Retrospective cohort study	Multi-centre	2015–2017	India	Tertiary	Not described	199	Blood (*N* = 126) Non-blood (*N* = 73)
Soldini, 2018	Retrospective cohort study	Single-centre	2005–2015	Italy	Tertiary	Adult	190	Blood
Sun, 2019	Retrospective cohort study	Single-centre	1 March 2012–28 February 2018	China	Tertiary	Adults and paediatric	323 *Candida* spp. 120/323 *C. parapsilosis*	Blood
Vitalis, 2020	Retrospective cohort study	Single-centre	January 2013–December 2018	Hungary	Tertiary	Not described	127 *Candida* spp. 23/127 *C. parapsilosis*	Blood
Wang, 2016	Other: laboratory surveillance study	Single-centre	2010–2014	China	Tertiary	Not described	443 yeasts (including non-*Candida* spp.) 212/443 *C. parapsilosis sensu stricto* 5/443 *C. metapsilosis*	Blood (*N* = 176) Vascular catheter tips (*N* = 33) Bronchoalveolar lavage (*N* = 2) Surgical drainage (*N* = 1)
Wu, 2020	Retrospective cohort study	Single-centre	2014–2018	China	Tertiary	Neonates	58	Blood
Wu, 2018	Retrospective cohort study	Single-centre	1 January 2010–31 December 2010	Taiwan	Tertiary and primary	Hospitalised patients >18 years old	270 *Candida* spp. 61/270 *C. parapsilosis*	Blood
Xiao, 2015	Other: prospective, laboratory-based sensitivity study	Multi-centre	August 2009–July 2012	China	Tertiary	Not described	1072 *Candida* spp. 392/1072 *C. parapsilosis* complex 325/392 *C. parapsilosis sensu stricto*	Invasive isolates (for *C. parapsilosis*) Blood 58% (228/392) Ascitic fluid 15.1% (59/392) CVC 10.7% (42/392) Pus 6.6% (26/392) Other mixed for remainder including 5 CSF and 10 peritoneal dialysis fluid
Zhang, 2020	Retrospective cohort study	Multi-centre	August 2016–July 2017	China	Not stated	Adult and paediatric	319	Blood 230 Abdominal *Candida* infection 61 CNS infection 6 Other invasive 22
Zhang, 2020	Retrospective cohort study	Single-centre	January 2012–December 2018	China	Tertiary	Adult >14 years old	236 total—subdivided into 172 (post-surgery) 74/172 *C. parapsilosis*	Blood
Zhou, 2019	Retrospective cohort study	Single-centre	January 2012–December 2017	China	Tertiary	Patients with major burns	39 total 11/39 *C. parapsilosis*	Blood

Data on azole susceptibility and susceptibility to other antifungal agents are presented in Tables 5 and 6. Most breakpoint interpretations for organism susceptibility utilised guidelines published by the Clinical and Laboratory Standards Institute (CLSI) or the European Committee on Antimicrobial Susceptibility Testing (EUCAST). Given the propensity for *C. parapsilosis* to form biofilm on foreign material, one study looked at antifungal susceptibility, specifically at different biofilm loads, to ascertain if there was decreased susceptibility.^9^ One study only provided susceptibility data for *C. albicans* and NAC, but no specific data for *C. parapsilosis*.^31^

**Table 5.** Drug susceptibility—azole agents.

Author, year	MIC determination method	Number of isolates	Fluconazole	Itraconazole	Posaconazole	Voriconazole
Arastehfar, 2020	CLSI M27-A3 with CLSI CBPs and ECVs	94	GM: 0.3425 Range: 0.06–16 MIC50: 0.25 MIC90: 1	GM: 0.0903 Range: 0.015–2 MIC50: 0.125 MIC90: 0.25	NA	GM: 0.0154 Range: <0.015–0.5 MIC50: 0.015 MIC90: 0.06
Asadzadeh, 2019	Etest, CLSI CBPs	88	Range: 0.016–3 MIC50: 0.19 MIC90: 0.75	NA	NA	NA
Asadzadeh, 2017	Etest, Vitek2 YST AST, CLSI Broth microdilution for fluconazole resistant isolates; CLSI CBP	442	*Candida parapsilosis sensu stricto* S: 425/442 (96.2%) SDD: 2/442 (0.5%) R: 15/442 (3.4%)	NA	NA	NA
Ataides, 2015	Etest	78	Range: 0.064–4 MIC50: 1 MIC90: 2 SDD: 2.5% R: 0%	Range: 0.002–2 MIC50: 0.125 MIC90: 0.125 SDD: 6.4% R: 1.3%	Range: 0.003–0.094 MIC50: 0.016 MIC90: 0.032 R: 0%	Range: 0.002–0.25 MIC50: 0.012 MIC90: 0.047 R: 0%
Barchiesi, 2016	Sensitre YeastOne, CLSI CBPs	63	Range: ≤0.12–4 MIC50: 0.25 MIC90: 1 S: 98.4% SDD: 1.6% R: 0%	Range: ≤0.015–0.25 MIC50: 0.06 MIC90: 0.12 WT: 100%	Range: ≤0.008–0.12 MIC50: 0.03 MIC90: 0.06 WT: 100%	Range: ≤0.008–0.06 MIC50: ≤0.008 MIC90: 0.015 S: 100%
Boan, 2019	Sensititre YeastOne, CLSI CBPs	14	R: 1/14 (7%)	NA	NA	R: 0%
Caggiano, 2017	Sensititre YeastOne, CLSI CBPs	25	Range: 0.25–4 R: 0%	NA	NA	NA
Castanheira, 2017	CLSI M27-A3, CLSI CBPs	417	MIC50: 0.5 MIC90: 2 S: 95.7% R: 3.8%	NA	MIC50: 0.016 MIC90: 0.12	MIC50: 0.015 MIC90: 0.03 S: 96.4% R: 0.7%
Castanheira, 2020	CLSI M27-A3, CLSI CBPs and ECVs	431	R: 8.8% across Asia-Pacific, Europe, Latin America, and North America R: 15.1% in Europe	NA	NWT: 0.5%	R: 0.2%
Cattana, 2017	CLSI M27-A3, CLSI CBPs and ECVs	59	GM: 1.50 Range: 0.25–4 MIC50: 2 MIC90: 2 S: 91.5% SDD: 8.5% R: 0%	GM: 0.10 Range: ≤0.03–0.25 MIC50: ≤0.03 MIC90: ≤0.03 WT: 100%	NA	GM: 0.06 Range: ≤0.03–0.125 MIC50: ≤0.03 MIC90: 0.06 S: 100%
Chapman, 2015	Sensititre YeastOne, CLSI CBP and ECV	82	GM: 0.54 Range: <0.12–32 MIC90: 1 S: 98.8% R: 1.2%	GM: 0.039 Range: <0.015–0.5 MIC90: 0.12 S: 100% R: 0%	GM: 0.02 Range: <0.008–0.12 MIC90: 0.06 S: 100% R: 0%	GM: 0.01 Range: <0.008–1 MIC90: 0.03 S: 98.8% R: 1.2%
Chen, 2015	Sensitire YeastOne, CLSI CBPs and ECVs	323	*Candida parapsilsosis sensu stricto* Range: 0.25–4 MIC50: 0.5 MIC90: 1 S: 250/256 (98%) SDD: 6/256 (2%) R: 0% *Candida metapsilosis* Range: 0.5–16 MIC50: 1 MIC90:2 S: 30/33 (91%) R: 3/33 (9%) *C. orthopsilosis* Range: 0.25–2 MIC50: 0.5 MIC90: 1 S: 34/34 (100%)	*Candida parapsilsosis sensu stricto* Range: 0.03–0.5 MIC50: 0.06 MIC90: 0.12 WT: 256/256 (100%) *Candida metapsilosis* Range: ≤0.015–0.12 MIC50: 0.06 MIC90: 0.12 WT: 33/33 (100%) *Candida orthopsilosis* Range: 0.06–0.25 MI50: 0.12 MIC90: 0.25 WT: 34/34 (100%)	*Candida parapsilosis sensu stricto* Range: 0.015-0.25 MIC50: 0.03 MIC90: 0.06 WT: 255/256 (99.6%) NWT: 1/256 (0.3%) *Candida metapsilosis* Range: 0.015–0.06 MIC50: 0.03 MIC90: 0.06 WT: 33/33 (100%) *Candida orthopsilosis* Range: 0.03–0.12 MIC50: 0.06 MIC90: 0.12 WT: 34/34 (100%)	*Candida parapsilosis sensu stricto* Range: ≤0.008–0.06 MIC50: ≤0.008 MIC90: 0.015 S: 256/256 (100%) *Candida metapsilosis* Range ≤0.008–0.12 MIC50: 0.015 MIC90: 0.03 S: 33/33 (100%) *Candida orthopsilosis* Range: ≤0.008–0.06 MIC50: 0.015 MIC90: 0.03 S: 34/34 (100%)
Da Silva, 2015	CLSI M27-A3	81	*Candida parapsilosis sensu stricto* (77) • Planktonic GM: 1.0 Range: 0.24–4 MIC50: 0.5 MIC90: 2 • Sessile (biofilm) Range: 0.25–≥64 MIC50: ≥64 *Candida orthopsilosis* (*n* = 2) • Planktonic: Range 1–2 • Sessile (biofilm) Range: ≥64 *Candida metapsilosis* (*n* = 2) • Planktonic Range: 2–16	NA	NA	*Candida parapsilosis sensu strictu* (*n* = 77) • Planktonic: GM: 0.06 Range: ≤0.03–0.25 MIC50: ≤0.03 MIC90: 0.06 • Sessile (biofilm): Range: 4–≥16 MIC50: ≥16 *Candida orthopsilosis* (*n* = 2) • Planktonic: Range: ≤0.03–0.12 • Sessile (biofilm): Range: ≥16 *Candida metapsilosis* (*n* = 2) • Planktonic: Range: 0.06–0.5
Desnos-Ollivier, 2018	EUCAST	161	• French isolates R: 25/116 (22%) • Uruguay isolates R: 0/45 (0%)	NA	• French isolates R: 36/116 (31%) • Uruguay isolates not tested	• French isolates R: 10/116 (9%) • Uruguay isolates R: 0/45 (0%)
Ejaz, 2019	Sensititre YeastOne, CLSI CBPs	31	GM: 0.22 Range: 0.12–1 R: 0/31 (0%)	GM: 0.04 Range: 0.015–0.5 R: 0/31 (0%)	GM: 0.07 Range: 0.015–0.5 R: 0/31 (0%)	GM: 0.02 Range: 0.008–1 R: 0/31 (0%)
Fernandez-Ruiz, 2014	EUCAST	194	*Candida parapsilosis sensu stricto* GM: 0.48 MIC 90: 1 R: 9/180 (5%) *Candida orthopsilosis* GM: 0.5 R: 0/7 (0%) *Candida metapsilosis* GM: 1 R: 0/2 (0%)	NA	NA	*Candida parapsilosis sensu stricto* GM: 0.02 MIC90: 0.03 R: 0/180 (0%) *Candida orthopsilosis* GM: 0.02 R: 0/7 (0%) *Candida metapsilosis* GM: 0.03 R: 0/2 (0%)
Ghezzi, 2017	Sensititre YeastOne, CLSI BPs	189	Range: 0.12–25 MIC50: 0.5 MIC90: 4 R: 15/187 (8.56%)	Range: 0.015–2 MIC50: 0.06 MIC90: 0.25 NWT: 2/188 (1.06%)	Range: 0.008–2 MIC50: 0.03 MIC90: 0.125 NWT: 17/188 (9.04%)	Range: 0.008–2 MIC50: 0.008 MIC90: 0.12 R: 3/188 (1.60%)
Guo, 2017	CLSI M27-A3, CLSI CBPs	161	Range: 0.064–16 MIC50: 0.5 MIC90: 2 R: 1.9%	Range: 0.016–2 MIC50: 0.25 MIC90: 0.5 R: 0.6%	NA	Range: 0.016–1 MIC50: 0.016 MIC90: 0.064 R: 0.6%
Hilmioglu-Polat, 2018	CLSI M27-A3, CLSI CBPs	170	GM: 2.95 Range: 0.06–32 MIC50: 1 MIC90: 4 R: 9.1%	NA	GM: 0.1 Range: 0.008–0.5 MIC50: 0.063 MIC90: 0.25 R: Not determined	GM: 0.21 Range: 0.016–8 MIC50: 0.016 MIC90: 0.125 R: 4.5%
Khodavaisy, 2020	CLSI M27-A3, CLSI CBPs	101	*Candida parapsilosis* complex GM: 0.9772 Range: 0.125–16 MIC50: 1 MIC90: 2 S: 93.1% *Candida parapsilosis sensu stricto* GM: 0.9026 Range: 0.125–16 MIC50: 1 MIC90: 2 S: 93.2%	*Candida parapsilosis* complex GM: 0.2027 Range: 0.031–2 MIC50: 0.125 MIC90: 1 S: 89.1% *Candida parapsilosis sensu stricto* GM: 0.1805 Range: 0.03–2 MIC50: 0.125 MIC90: 0.5 S: 92%	*Candida parapsilosis* complex GM: 0.0175 Range: 0.016–0.125 MIC50: 0.016 MIC90: 0.016 S: 100% *Candida parapsilosis sensu stricto* GM: 0.0166 Range: 0.016–0.06 MIC50: 0.016 MIC90: 0.016 S: 100%	*Candida parapsilosis* complex GM: 0.0189 Range: <0.016–0.25 MIC50: 0.016 MIC90: 0.031 S: 99% *Candida parapsilosis sensu stricto* GM: 0.0185 Range: <0.016–0.125 MIC50: 0.016 MIC90: 0.03 S: 98.9%
Kontoyiannis, 2017	CLSI M27-A3, CLSI CBPs	58	Range: 0.25–128 MIC50: 0.5 MIC90: 16 S: 88.9%	NA	NA	Range: ≤0.015–1 MIC50: ≤0.03 MIC90: 0.5 S: 100%
Lin, 2015	Sensititre YeastOne	77	Range: 0.25–8 MIC50: 1 MIC90: 2 R: 3%	Range: 0.015–0.25 MIC50: 0.06 MIC90: 0.12	Range: 0.015–0.12 MIC50: 0.03 MIC90: 0.06	Range: 0.008–0.15 MIC50: 0.008 MIC90: 0.15
Lindberg, 2019	Sensititre YeastOne, EUCAST and CLSI CBPs	29	Range: 0.25–2 MIC50: 0.5 MIC90: 2 S: 100%	Range: 0.015–0.25 MIC50: 0.03 MIC90: 0.12 S: 97%	Range: 0.015–2 MIC50: 0.03 MIC90: :0.06 S: 97%	Range: 0.008–0.06 MIC50: 0.015 MC90: 0.03 S: 100%
Lotfali, 2016	CLSI M27-S3, CLSI CBPs	120	R: 3/120 (2.5%)	R: 3/120 (2.5%)	NA	NA
Martini, 2020	CLSI M27-A3, CLSI CBPs	241	Range: 8–128 SDD: 1/241 (0.4%) R: 53/241 (22%)	NA	NA	Range: 1–2 SDD: 22/241 (9.1%) R: 27/241 (11.2%)
Medeiros, 2019	CLSI M27-A3, CLSI CBPs	9	Range: 0.125–16 MIC50: 0.5 R: 1/9 (11.1%)	Range: <0.03–0.03 MIC50: <0.03 R: 0/9 (0%)	NA	NA
Mesini, 2020	Sensititre YeastOne, CLSI CBPs	694 -1/3 available for testing	R: 64/194 (33%)	NA	NA	R: 34/187 (18.2%)
Pharkjaksu, 2018	Sensititre YeastOne, CLSI CBPs	139	GM: 0.923 Range: ≤0.12–≥256 MIC50: 0.5 MIC90: 4 R: 4/66 (6.06%)	GM: 0.077 Range: ≤0015–0.5 MIC50: 0.06 MIC90: 0.25	GM: 0.046 Range: 0.008–0.5 MIC50: 0.06 MIC90:0.12	GM: 0.025 Range: ≤0.008–≥8 MIC50: 0.015 MIC90: 0.12 R: 3/66 (4.55%)
Pinhati, 2016	VITEK 2 YST AST (28/28 isolates) and CLSI M27-A3 (14/28 isolates)	28	Vitek 2: SDD or R: 21/28 (75%) CLSI: SDD or R: 11/14 (78.6%)	NA	NA	NA
Sadeghi, 2018	Not stated	79	R: 4.5%	NA	NA	NA
Singh, 2019	CLSI M27-A3, CLSI CBPs	199	Range: 0.125–32 R: 28%	Range: ≤0.03–0.5	Range: ≤0.03–0.25	Range: ≤0.03–1
Soldini, 2018	Planktonic MIC: CLSI M27-A3 Sessile MIC: XTT reduction assay 30 isolates: 10 low biofilm, 10 moderate biofilm, 10 high biofilm forming	30	LBF: planktonic R: 0% sessile R: 0% MBF: planktonic R: 0% sessile R: 10% HBF: planktonic R: 0% sessile R: 80%	NA	NA	LBF: planktonic R: 0% sessile R: 20% MBF: planktonic R: 0% sessile R: 100% HBF: planktonic R: 0% sessile R: 100%
Sun, 2019	CLSI M27-A2. Breakpoints not defined.	120	Range: 0.125–1 MIC50: 0.125 MIC90: 0.25 R: 0%	Range: 0.125–0.25 MIC50: 0.125 MIC90: 0.125 R: 0%	NA	NA
Wang, 2016	CLSI M44-A2 Disc diffusion, CLSI CBPs	212	R: 0%	NA	NA	R: 0%
Wu, 2020	CLSI M27-A, EUCAST CBPs and CLSI CBPs	58	Range: <1 R: 0%	Range: <0.125	NA	Range: <0.062
Wu, 2018	Sensititre YeastOne, CLSI CBPs	61	Range: ≤0.12–4 MIC50: 0.5 MIC90: 2 SDD: 5.4% R: 0%	Range: ≤0.015–0.25 MIC50: 0.06 MIC90 :0.12	Range: 0.015–0.06 MIC50: 0.03 MIC90: 0.06	Range: ≤0.008–0.06 MIC50: ≤0.008 MIC90: 0.03 R: 0%
Xiao, 2015	Sensititre YeastOne, CLSI CBPs. CLSI M44-A2 Disc diffusion also used for fluconazole and voriconazole	392 total	*Candida parapsilosis* complex Range: ≤0.12–128 R: 6/392 (1.5%) *Candida parapsilosis sensu stricto* Range: ≤0.12–16 R: 5/325 (1.5%)	*Candida parapsilosis* complex Range: ≤0.015–0.25 R: 0/392 (0%) *Candida parapsilosis sensu stricto* Range: ≤0.015–0.25 R: 0%	*Candida parapsilosis* complex Range: ≤0.008–0.25 R: 0/392 (0%) *Candida parapsilosis sensu stricto* Range: ≤0.08–0.25 R: 0/325 (0%)	*Candida parapsilosis* complex Range: ≤0.008–0.5 R: 0/392 (0%) *Candida parapsilosis sensu stricto* Range: ≤0.008–0.5 R: 0/325 (0%)
Zhang, 2020	Sensititre YeastOne, CLSI CBPs or ECVs	319	Range: ≤0.12–128 MIC50: 0.5 MIC90: 1 R: 5%	Range: ≤0.015–2 MIC50: 0.06 MIC90: 0.12 NWT: 0%	Range: ≤0.008–2 MIC50: 0.03 MIC90: 0.12 NWT: 2.5%	Range: ≤0.08–4 MIC50: 0.015 MIC90: 0.06 R: 4.7%
Zhang, 2020	ATB FUNGUS 3, CLSI or EUCAST CBPs	74	Range: ≤0.5–8 MIC50: 1 MIC90: 4 R: 1.4%	Range: ≤0.06–0.25 MIC50: 0.125 MIC90: 0.125 R: 2.7%	NA	Range: ≤0.03–0.25 MIC50: 0.06 MIC90: 0.125 R: 0%
Zhou, 2019	Disk diffusion using Neosensitabs, CLSI CBPs	11	R: 10% (approx.)	R: 0%	NA	R: 0%

Note: Susceptibility values are expressed as minimum inhibitory concentrations (MICs) in mg/l (EUCAST) or mg/ml (CLSI). GM, Geometric mean; MIC50, MIC of 50% of isolates; MIC90, MIC of 90% of isolates; S, susceptible; SDD, susceptible dose dependent; I, intermediate; R, resistant; WT, wild type; NWT, non-wild type; NA, not available. Data and interpretive breakpoints are presented here as reported in source documents.

**Table 6.** Drug susceptibility—other antifungal agents.

Author, year	MIC determination method	Number of isolates	Anidulafungin	Caspofungin	Micafungin	Amphotericin B	Flucytosine
Arastehfar, 2020	CLSI M27-A3 with CLSI CBPs and ECVs	94	GM: 1.1263 Range: 0.25–4 MIC50: 1 MIC90: 2	NA	GM: 0.7095 Range: 0.25–8 MIC50: 0.5 MIC90: 2	GM: 0.3674 Range: 0.03–2 MIC50: 0.5 MIC90: 1	NA
Asadzadeh, 2019	Etest, CLSI CBPs	88	NA	Range: 0.016–0.75 MIC50: 0.25 MIC90: 0.5	NA	Range: 0.002–0.19 MIC50: 0.0125 MIC90: 0.047	NA
Ataides, 2015	Etest	78	NA	Range: 0.125–32 MIC50: 0.75 MIC90: 16 SDD: 3.8% R: 10.2%	NA	Range: 0.064–32 MIC50: 0.5 MIC90: 1 R: 1.3%	NA
Barchiesi, 2016	Sensitre YeastOne, CLSI CBPs	63	Range: 0.12–2 MIC50: 0.5 MIC90: 2 S: 100%	Range: 0.06–1 MIC50: 0.25 MIC90: 0.5 S: 100%	Range: 0.12–2 MIC50: 1 MIC90: 2 S: 100%	Range: ≤0.12–1 MIC50: 0.5 MIC90: 0.5 WT: 100%	Range: ≤0.06–1 MIC50: ≤0.06 MIC90: 0.25 WT: 97%
Boan, 2019	Sensititre YeastOne CLSI CBPs	14	NA	R: 0%	NA	NA	NA
Caggiano, 2017	Sensititre YeastOne, CLSI CBPs	25	Range: 0.5–2	Range: 0.12–0.5	Range: 0.5–2	Range: 0.06–0.5	NA
Castanheira, 2017	CLSI M27-A3, CLSI CBPs and ECVs	417	MIC50: 2 MIC90: 4 S: 88.7% R: 0%	MIC50: 0.25 MIC90: 0.5 S: 100% R: 0%	MIC50: 1 MIC90: 2 S: 100% R: 0%	MIC50: 1 MIC90: 1 WT: 100%	NA
Castanheira, 2020	CLSI M27-A3, CLSI CBPs and ECVs	431	I: 6.5% R: 0%	R: 0%	R: 0%	NWT: 0%	NA
Cattana, 2017	CLSI M27-A3, CLSI CBPs and ECVs	59	GM 1.10 Range: 0.015–4.00 MIC50: 1 MIC90: 2 S: 97% I: 3%	GM 1.00 Range: 0.125–2.00 MIC50: 1 MIC90: 1 S: 100%	NA	GM 1.00 Range: 0.50–2.00 MIC50: 1 MIC90: 2 WT: 100%	NA
Chapman, 2016	Sensititre YeastOne, CLSI CBP and ECV	82	GM: 0.72 Range: 0.12–2 MIC90: 2 S: 100% R: 0%	GM: 0.24 Range: 0.06–1 MIC90: 0.50 S: 100% R: 0%	GM: 0.75 Range: 0.12–4 MIC90: 2 S: 98.8% SDD: 1.2% R: 0%	GM: 0.44 Range: 0.12–2 MIC90: 0.5 S: 100% R: 0%	GM: 0.06 Range: <0.06–0.5 MIC90: 0.12 S: 100% R: 0%
Chen, 2015	Sensititre YeastOne, CLSI CBPs and ECVs	323	*Candida parapsilosis sensu stricto* Range: 0.5–4 MIC50: 1 MIC90: 2 S: 252/256 (98%) I: 4/256 (2%) *Candida metapsilosis* Range: 0.12–1 MIC50: 0.5 MIC90: 0.5 S: 33/33 (100%) *Candida orthopsilosis* Range: 0.5–2 MIC50: 1 MIC90: 1 S: 34/34 (100%)	NA	*Candida parapsilosis sensu stricto* Range: 0.25–8 MIC50: 1 MIC90: 2 S: 252 (98%) I: 3 (1%) R: 1 (0.3%) *Candida metapsilosis* Range: 0.25–1 MIC50: 0.5 MIC90: 0.5 S: 33/33 (100%) *Candida orthopsilosis* Range: 0.25–1 MIC50: 0.5 MIC90: 1 S: 34/34 (100%)	*Candida parapsilosis sensu stricto* Range: ≤0.12–1 MIC50: 0.5 MIC90: 0.5 WT: 256/256 (100%) *Candida metapsilosis* Range: ≤0.12–1 MIC50: 0.5 MIC90: 0.5 WT: 33/33 (100%) *Candida orthopsilosis* Range: 0.25–1 MIC50: 0.5 MIC90: 0.5 WT: 34/34 (100%)	*Candida parapsilosis sensu stricto* Range: ≤0.06–>64 MIC50: 0.12 MIC90: 0.25 WT: 255/256 (99.6%) NWT: 1(0.3%) *Candida metapsilosis* Range: ≤0.06–>64 MIC50: ≤0.06 MIC90: ≤0.06 WT: 32/33 (97%) NWT: 1 (3%) *Candida orthopsilosis* Range: ≤0.06–>64 MIC50: 0.12 MIC90: 0.12 WT: 33/34 (97%) NWT: 1/34 (3%)
Da Silva, 2015	CLSI M27-A3	81	NA	*Candida parapsilosis sensu strictu* (*n* = 77) • Planktonic GM: 0.37 Range: 0.03–2 MIC50: 1 MIC90: 2 • Sessile Range: 1–≥8 MIC50: ≥ 8 *Candida orthopsilosis* (*n* = 2) • Planktonic Range: 0.25–0.5 • Sessile Range: ≥8 *Candida metapsilosis* (*n* = 2) • Planktonic Range: 0.5–1	NA	*Candida parapsilosis sensu strictu* (*n* = 77) • Planktonic GM: 0.35 Range: 0.12–1 MIC50: 0.5 MIC90: 1 • Sessile Range: 1– ≥16 MIC50: ≥16 *Candida orthopsilosis* (*n* = 2) • Planktonic Range: 1 • Sessile Range: ≥16 *Candida metapsilosis* (*n* = 2) • Planktonic Range: 0.25–0.5	NA
Devrim, 2016	Etest, CLSI CBPs	43/161	NA	Range: 0.5–2 MIC50: 1 MIC90: 2	NA	NA	NA
Ejaz, 2019	Sensititre YeastOne, CLSI BPs	31	GM: 0.04 Range: 0.015–0.12 R: 0/31 (0%)	GM: 0.08 Range: 0.015–0.12 R: 0/31 (0%)	GM: 0.03 Range: 0.008–0.25 R: 0/31 (0%)	GM 0.20 Range: 0.12–1 R: 0/31 (0%)	GM: 0.63 Range: 0.06–64 R: 2/31 (6.5%)
Fernandez-Ruiz, 2014	EUCAST	194	*Candida parapsilosis sensu stricto* (180) GM: 0.93 MIC90: 2.0 R: 0/180 (0%) *Candida orthopsilosis* GM: 0.37 R: 0/7 (0%) *Candida metapsilosis* GM: 0.12 R: 0/2 (0%)	NA	*Candida parapsilosis sensu stricto* (180) GM: 0.84 MIC90: 2.0 R: 2/180 (1.1%) *Candida orthopsilosis* GM: 0.30 R: 0/7 (0%) *Candida metapsilosis* (2) GM: 0.25 R: 0/2 (0%)	NA	NA
Ghezzi, 2017	Sensititre YeastOne, CLSI BPs	189 total	Range: 0.015–8 MIC50: 0.5 MIC90: 2 R: 1/173 (0.58%)	Range: 0.015–2 MIC50: 0.25 MIC90: 0.5 R: 0/188 (0%)	Range: 0.015–4 MIC50: 0.5 MIC90: 2 R: 0/184 (0%)	Range: 0.12–1 MIC50: 0.25 MIC90: 1 NWT: 0/189 (0%)	Range: 0.06–16 MIC50: 0.06 MIC90: 0.25 NWT: 5/177 (2.82%)
Guo, 2017	CLSI M27-A3, CLSI CBPs	161	NA	Range: 0.008–4 MIC50: 0.5 MIC90: 2 R: 0%	Range: 0.008–4 MIC50: 0.5 MIC90: 2 R: 0%	Range: 0.016–1 MIC50: 0.5 MIC90: 0.5 R: 0%	Range: 0.064–0.5 MIC50: 0.064 MIC90: 0.125 R: 0%
Hilmioglu-Polat, 2018	CLSI M27-A3, CLSI CBPs	170	GM: 0.68 Range: 0.016–2 MIC50: 1 MIC90:1 R: 0%	GM: 0.38 Range: 0.016–1 MIC50: 0.5 MIC90: 0.5 R: 0%	NA	GM: 0.61 Range: 0.25–2 MIC50: 0.5 MIC90: 1	NA
Khodavaisy, 2020	CLSI M27-A3, CLSI CBPs	101	*Candida parapsilosis* GM: 0.2771 Range: 0.008–4 MIC50: 0.25 MIC90: 1 S: 98% *Candida parapsilosis sensu stricto* GM: 0.2904 Range: 0.008–4 MIC50: 0.25 MIC90: 1 S: 97.7%	NA	*Candida parapsilosis* GM: 0.1711 Range: 0.008–8 MIC50: 0.25 MIC90: 0.5 S: 99% *Candida parapsilosis sensu stricto* GM: 0.1988 Range: 0.008–8 MIC50: 0.25 MIC90: 1 S: 98.9%	*Candida parapsilosis* GM: 0.0455 Range: 0.016–2 MIC50: 0.016 MIC90: 0.5 S: 100% *Candida parapsilosis sensu stricto* GM: 0.0462 Range: 0.016–2 MIC50: 0.016 MIC90: 1 S: 100%	NA
Kontoyiannis, 2017	CLSI M27-A3, CLSI CBPs	58	Range: ≤0.03–16 MIC50: 1 MIC90: 4 S: 86.2%	NA	NA	NA	NA
Lin, 2015	Sensititre YeastOne	77	Range: 0.12–2 MIC50: 1 MIC90: 2	Range: 0.25–1 MIC50: 0.5 MIC90: 1	Range: 0.25–4 MIC50: 1 MIC90: 2	Range: 0.25–1 MIC50: 1 MIC90: 1	NA
Lindberg, 2019	Sensititre YeastOne, CLSI and EUCAST CBPs	29	Range: 0.25–2 MIC50: 0.5 MIC90: 2 S: 100%	Range: 0.12–1 MIC50: 0.25 MIC90: 0.5 S: 100%	Range: 0.5–2 MIC50: 1 MIC90: 2 S: 100%	Range: 0.12–1 MIC50: 0.25 MIC90: 0.5 S: 100%	Range: 0.06–0.25 MIC50: 0.06 MIC90: 0.06
Lotfali, 2016	CLSI M27-S3, CLSI CBPs	120	NA	NA	NA	R: 2/120 (1.6%)	NA
Lovero, 2016	Various methods used for purpose of comparison. CLSI broth microdilution data shown here.	163	*Candida parapsilosis sensu stricto* Range: 0.5–2 MIC50: 1 MIC90: 2 *Candida orthopsilosis* Range: 0.5–2 MIC50: 1 MIC90: 2	*Candida parapsilosis sensu stricto* Range: 0.25–4 MIC50: 2 MIC90: 2 *Candida orthopsilosis* Range: 0.25–2 MIC50: 1 MIC90: 2	*Candida parapsilosis sensu stricto* Range: 0.008–2 MIC50: 0.5 MIC90: 2 *Candida orthopsilosis* Range: 0.008–2 MIC50: 0.5 MIC90: 2	NA	NA
Medeiros, 2019	CLSI M27-A3, CLSI CBPs	9	NA	NA	Range: <0.015–0.06 MIC50: 0.03 R: 0%	Range: 0.125–1.0 MIC50: 0.5 R: 0%	NA
Pharkjaksu, 2018	Sensititre YeastOne, CLSI BPs	66/139 *Candida parapsilosis sensu stricto*	Range: 0.015–4 MIC50: 1 MIC90: 2 R: 0%	GM: 0.464 Range: 0.03–2 MIC50: 0.5 MIC90: 1 R: 0%	GM: 0.842 Range: 0.015–4 MIC50: 1 MIC90: 2 R: 0%	GM: 0.742 Range: ≤0.12–4 MIC50: 0.5 MIC90: 2 R: 1/66 (1.52%)	GM: 0.097 Range: ≤0.06–0.25 MIC50: 0.12 MIC90: 0.12
Singh, 2019	CLSI M27-A3 CLSI CBPs	199	NA	NA	NA	Range: ≤0.03–1	Range: 0.125–0.5
Soldini, 2018	Planktonic MIC: CLSI broth microdilution Sessile MIC: XTT reduction assay 30 isolates: 10 low biofilm, 10 moderate biofilm, 10 high biofilm forming	30	LBF: planktonic R: 0% Sessile R: 0% MBF: planktonic R: 0% Sessile R: 0% HBF: planktonic R: 0% Sessile R: 60%	NA	NA	LBF: planktonic MIC R: 0 sessile R: 0% MBF: planktonic MIC R: 0 sessile R: 80% HBF: planktonic R: 0% sessile R: 40%	NA
Sun, 2019	CLSI M27-A2 Breakpoints not defined	120	NA	NA	NA	Range: 0.5–1 MIC50: 0.5 MIC90: 0.5	Range: 0.125–1 MIC50: 0.125 MIC90: 0.25 R: 0%
Wu, 2020	CLSI M27-A, EUCAST CBPs and CLSI CBPs	58	NA	NA	Range: <2	Range: <0.5	Range: <4
Wu, 2018	Sensititre YeastOne CLSI CBPs	61	Range: 0.12–2 MIC50: 1 MIC90: 2 R: 0%	Range: 0.12–1 MIC50: 0.5 MIC90: 1 R: 0%	Range: 0.12–2 MIC50: 1 MIC90: 1 R: 0%	Range: 0.25–8 MIC50: 0.5 MIC90: 0.5	Range: ≤0.06–>64 MIC50: 0.12 MIC90: 0.25
Xiao, 2015	Sensititre YeastOne CLSI CBPs CLSI M44-A2 Disc diffusion (used for fluconazole and voriconazole)	392	*Candida parapsilosis complex* Range: 0.015–2 R: 0% *Candida parapsilosis sensu stricto* Range: 0.12–2 R: 0%	*Candida parapsilosis complex* Range: 0.03–2 R: 0% *Candida parapsilosis sensu stricto* MIC 0.06–2 R: 0%	*Candida parapsilosis complex* Range: 0.015–2 R: 0% *Candida parapsilosis sensu stricto* Range: 0.06–2 R: 0%	*Candida parapsilosis complex* Range: ≤0.12–2 R: 0% *Candida parapsilosis sensu stricto* Range: ≤0.12–2 R: 0%	*Candida parapsilosis complex* Range: ≤0.06–1 R: 0.8% *Candida parapsilosis sensu stricto* Range: ≤0.06–1 R: 2.3%
Zhang, 2020	Sensititre YeastOne, CLSI CBPs or ECVs	319	Range: 0.03–2 MIC50: 1 MIC90: 2 R: 0%	Range: 0.12–2 MIC50: 0.5 MIC90: 1 R: 0%	Range: ≤0.008–4 MIC50: 1 MIC90: 2 R: 0%	Range: ≤0.12–2 MIC50: 0.5 MIC90: 1 NWT: 0%	Range: ≤0.06–>64 MIC50: ≤0.06 MIC90: 64 NWT: 11.6%
Zhang, 2020	ATB FUNGUS 3, CLSI or EUCAST CBPs	74	NA	NA	NA	Range: ≤0.25–1 MIC50: 0.5 MIC90: 0.5 R: 0%	NA
Zhou, 2019	Disk diffusion using Neosensitabs, CLSI CBPs	11	NA	R: 0%	R: 0%	R: 0%	NA

Note: Susceptibility values are expressed as minimum inhibitory concentrations (MICs) in mg/l (EUCAST) or mg/ml (CLSI). GM, geometric mean; MIC50, MIC of 50% of isolates; MIC90, MIC of 90% of isolates; S, susceptible; SDD, susceptible dose dependent; I, intermediate; R, resistant; WT, wild type; NWT, non-wild type; NA, not available. Data are given as provided in source documents.

**Table 7.** Risk factors.

Author, year	Study type	Study design	Study period	Country	Level of care	Population description	Number of patients	Risk factors	Relative association
Chen, 2015	Retrospective cohort study	Single-centre	2000–2012	Taiwan	Tertiary	Adults >18	323	Shock Antifungal therapy Central catheter removal Abdominal surgery	Risk not quantified
Devrim, 2016	Retrospective cohort study	Single-centre	December 2011–December 2013	Turkey	Tertiary paediatric intensive care	Paediatric	53	Prolonged CVC use	Risk not quantified
Fernandez-Ruiz, 2014	Other: sub-analysis of completed prospective, multi-centre, population-based surveillance programme	Multi-centre	May 2010–April 2011	Spain	Mixed	Mixed adult, paediatric and neonatal	773 episodes in >190 patients (*C. parapsilosis sensu stricto* only)	Risk of death: Orotracheal intubation at diagnosis of bloodstream infectionSeptic shock	Intubation adjusted: OR 2.81, 95% CI 1.19–6.65, *P* = .018 Septic shock: OR 2.91, 95% CI 0.88–9.64, *P* = .081
Guo, 2017	Other: prospective surveillance study	Multi-centre	January 2012–December 2013	China	Tertiary	Mixed adult and paediatric	1201 in total 161/1201—*Candida parapsilosis* complex	Chronic renal disease	Risk not quantified
Kontoyiannis, 2017	Other: pooled analysis—Pfizer study	Multi-centre	Not stated	Not stated	Not stated	Adult ≥18	70	Broad spectrum antibiotics	Risk not quantified
Lin, 2015	Retrospective cohort study	Single-centre	June 2008–June 2012	Taiwan	Tertiary	Mixed adult and paediatric	77	Risk of death: age <18 Onset of candidaemia in ICU Any malignancymetastatic solid tumour	Age <18: • OR 0.39, 95% CI: 0.09–1.79, *P* = .227 Onset of candidaemia in ICU • OR 0.77, 95% CI 0.13–4.39, *P =* .767 Any malignancy • OR 4.08, 95% CI 1.03–16.14, *P =* .045 Metastatic solid tumour • OR 1.72, 95% CI 0.46–6.45, *P* = .419
Medeiros, 2019	Retrospective cohort study	Single-centre	January 2011–December 2016	Brazil	Tertiary	Adult (+1 child)	68	For 30-day mortality all *Candida* species: Older age Severe sepsis Septic shock Blood pressure (hypotension vs. hypertension) Mechanical ventilation on candidaemia onset	For 30-day mortality, all *Candida* species: Older age • univariate logistic regression: *P* = .022, OR 1.041 (95% CI 1.006–1.078) • multivariate logistic regression: *P* = .040, OR 1.055 (95% CI 1.003–1.110) Severe sepsis • univariate logistic regression *P* < .001, OR 8.571 (95% CI 2.675–27.470) • multivariate logistic regression *P* = .009, OR 9.872, (95% CI 1.776–54.880) Septic shock • univariate: *P* = .035, OR 3.792 (95% CI 1.095–13.219) • multivariate: *P* = .558, OR 0.451 (95% CI 0.032–6.462) Blood pressure (hypotension vs. hypertension) • univariate: *P* = .003, OR 9.120 (95 CI 2.172–38.296) • multivariate; *P* = .031, OR 21.042 (95% CI 1.318–336.004) Mechanical ventilation on candidaemia onset • univariate: *P* = .009, OR 8.167 (95% CI 1.684–39.598) • multivariate *P* = .353, OR 2.613 (95% CI 0.344–19.869)
Mesini, 2020	Other: prospective cohort with retrospective cohort inclusion	Single-centre	2008–20112012–2016	Italy	Tertiary	Adult	660	Independent predictors *C. parapsilosis* vs. non-*C. parapsilosis*: time to infection Previous use of echinocandin Year in which episode registered Age Ward type CVC Solid tumour Previous treatment with steroids Charlson Comorbidity Index	Multivariate analyses age: OR 0.989, 95% CI: 0.979–1.000, *P* = .058 Time of infection: OR 1.008, 95% 1.002–1.014, *P* = .007 Previous use of echinocandin: OR 3.235, 95% CI 1.7008–6.127, *P* < .001 Year of candidaemia: *P* < .001 2012 no OR 2013 OR 1.027, 95% CI 0.512–2.058 2014 OR 1.391 95% CI 0.728–2.660 2015 OR 2.167, 95% CI 1.189–3.949 2016 OR 3.291, 95% CI 1.833–5.909 Univariate analyses Ward type: • rehab ward OR 3.089, 95% CI 0.922–10.347 • ICU OR 1.821, 95% CI 1.212–2.736 • surgical ward OR 1.054, 95% CI 0.691–1.609 Peripherally Inserted Central Catheter (PICC) OR 1.816, 95% CI 1.098–2.005 CVC non-PICC OR 1.021, 95% CI 0.705–1.480 Solid tumour: OR 0.722, 95% CI 0.489–1.066, *P* = .101 Steroid treatment: OR 0.718, 95% CI 0.444–1.160, *P* = .0176 Charlson Index: OR 0.929, 95% CI 0.858–1.005, *P* = .067
Pinhati, 2016	Cross sectional study	Single-centre	July 2011–February 2012	Brazil	Tertiary	Adults >/= 18	40	Diabetes—risk factor for fluconazole resistant *C. parapsilosis* Parenteral nutrition	Diabetes mellitus • univariate analysis: *P* = .013 • multivariate analysis: *P* = .001, OR 1.3–3.5 Parental nutritionunivariate analysis *P* = .059
Soldini, 2018	Retrospective cohort study	Single-centre	2005–2015	Italy	Tertiary	Adult	190	Risk for death: HBF/MBF *Candida parapsilosis* infection Presentation with septic shock	Multivariate regression analysis HBF/MBF *C. parapsilosis* infection: • OR 3.85, 95% CI 1.88–7.87 Septic shock: • OR 3.78, 95% CI 1.63–8.75
Sun, 2019	Retrospective cohort study	Single-centre	1 March 2012–28 February 2018	China	Tertiary	Adults and paediatric	323	Parenteral nutrition was a factor that independently predicted *C. parapsilosis* candidaemia	OR 0.183, 95% CI 0.098–0.340, *P* < .001
Wu, 2018	Retrospective cohort study	Single-centre	1 January 2010–31 December 2010	Taiwan	Tertiary and primary	Hospitalised patients >18 years old	253	• For *C. parapsilosis* infection: Antifungal agent exposure • For 30-day mortality from *C. parapsilosis* infection: SOFA (disease severity) score	Antifungal exposure (multivariate analysis): OR 7.261, 95% CI 1.603–32.879, *P* = .015 SOFA score (multivariate analysis): OR 0.225, 95% CI 1.002–1.573, *P* = .048

**Table 8.** Preventative measures.

Author, year	Study type	Study design	Study period	Country	Level of care	Population description	Number of patients	Preventative measures	Effectiveness
Cala, 2020	Retrospective cohort study	Single-centre	January 2015–December 2016	Italy	Tertiary	Hospitalised patients admitted to five different wards	70	Nosocomial transmission (four clusters found in four out of five wards, CP_P41 on General Surgery ward involved 53% of all isolates on this ward) Microsatellite genotyping could detect and prevent nosocomial spread	Not determined
Escribano, 2018	Retrospective cohort study	Single-centre	January 2007–December 2014 (in 2 periods) 2007–2010 (period 1) 2011–2014 (period 2)	Spain	Tertiary	Adults, paediatric and neonatal	432 in total *Candida parapsilosis* (*N* = 156) *Candida albicans* (*N* = 276)	Education package to reduce catheter related infections—not implemented as part of study	Incidence of catheter related infections decreased (all *C. albicans* and *C. parapsilosis*) from 0.53/1000 admissions per year (period 1) to 0.36/1000 admissions per year (period 2)
Fernandez-Ruiz, 2014	Other: sub-analysis of completed prospective, multi-centre, population-based surveillance programme	Multi-centre	May 2010–April 2011	Spain	Mixed	Mixed adult, paediatric and neonatal	773 episodes in >190 patients (*C. parapsilosis sensu stricto* only)	Early CVC removal	Adjusted OR 0.43; 95% CI 0.19–0.96; *P* = .040
Lin, 2015	Retrospective cohort study	Single-centre	June 2008–June 2012	Taiwan	Tertiary	Mixed adult and paediatric	77	Central venous catheter removal	Nil effect on mortality univariate analysis: survival 33.8% (26/77) vs. 33.8% (26/77) *P* = .410
Soldini, 2018	Retrospective cohort study	Single-centre	2005–2015	Italy	Tertiary	Adult	190	Receipt of adequate antifungal therapy ≤8 h CVC removal ≤48 h	Multivariate regression analysis Adequate antifungal therapy ≤48 h: OR 0.26, 95% CI 0.13–0.54 CVC removal ≤48 h: OR 0.21, 95% CI 0.10–0.45
Sun, 2019	Retrospective cohort study	Single-centre	1 March 2012–28 February 2018	China	Tertiary	Adults and paediatric	323	Removal of CVC <72 h	Multivariate analysis OR 0.248 (95% CI 0.067–0.915) *P* = .036

Susceptibility testing for azole antifungals was reported in 39 studies. Rates of resistance to fluconazole ranged from 0% to 33%. The extensive multi-centre global surveys included isolates collected between 2014 and 2017 and reported fluconazole resistance rates ranging from 3.3% to 8.8%.^34,35^ Although the reported rates of resistance in these studies increased between earlier and later study periods, the studies are not directly comparable, making a formal analysis of trends impossible. Several single-centre studies reported 0% resistance to fluconazole.^10,17,20,28,30,32,36^ In two single-centre studies from Italy, higher rates of resistance to fluconazole were reported (22% and 33%, respectively). These higher rates are potentially due to clonal transmission of fluconazole-resistant isolates within the same centre.^3,37^ A single-centre study from Brazil reported 75% resistance to fluconazole; however, this was a composite of isolates: susceptible dose-dependent (SDD) and resistant isolates from an outbreak in an ICU, and the study did not provide details on the proportions under each category.^8^ Fluconazole resistance rates were 1.9% (China), 2.5%–3.4% (Iran), 3.4% (Kuwait), 5% (Spain), 7% (Australia), and 28% (India) in multi-centre studies,^10,24,27,38–41^ and 3.8% and 8.8% in two global studies,^34,35^ respectively. The multi-centre study from India that reported a resistance rate to fluconazole of 28% identified that clonal strains of non-susceptible isolates were circulating in seven of eight Indian hospitals using molecular typing methods.^41^ A study from Kuwait reported an increase in fluconazole resistance for *C. parapsilosis* over a 3-year study period between 2012 and 2015.^38^

One study assessing the role of biofilm for antifungal susceptibility reported 0% fluconazole resistance for the planktonic form of *C. parapsilosis* but very high resistance (80%) when testing high biofilm mass and only 10% resistance for moderate biofilm mass. However, only 10 isolates with biofilm mass from each of high, moderate, and low biofilm-forming group were tested.^9^

Susceptibility to posaconazole and voriconazole was reported in 16 and 19 studies, with resistance rates ranging from 0% to 31% and 0% to 18.2%, respectively. Resistance rates of >10% were reported for posaconazole in one study using broth microdilution EUCAST method^36^ and in two for voriconazole.^3,37^ Only one study had susceptibility data for isavuconazole: For 199 isolates, the MIC range was ≤0.03–0.25 µg/ml.^41^ Only one study reported increasing rates of azole resistance over the study period.^3^ For the three studies with susceptibility data for *C. metapsilosis* and *C. orthopsilosis*, low-level fluconazole resistance is noted for *C. metapsilosis*. However, the number of isolates relative to *C. parapsilosis sensu stricto* was reasonably small in each study, and there was no specific pattern.^10,19,23^

Echinocandin susceptibility was reported in 30 studies (ranging from 9 to 433 isolates) with a mix of testing methods, interpretive criteria, and tested agents, as presented in Table 6. It should be noted that whilst caspofungin MICs are known to be unreliable, with both EUCAST and CLSI recommending not to perform or report for this agent, 23 of these 30 studies have provided results for caspofungin. There was virtually no echinocandin resistance, with rates reported between 0% and 1.1%, except for one study that only tested for caspofungin susceptibility and reported a 10.2% resistance rate.^17^ This study, however, utilised Etest strips as the method for which there are no validated interpretive criteria. Another study reported that 86.2% of isolates were susceptible to anidulafungin, but it is not reported how many isolates fell into the intermediate and resistant categories.^12^

A total of 28 studies reported amphotericin B susceptibility, and 6 reported 0% resistance rates.^21,25–27,33,42^ Only three studies showed 1.3%–1.6% rates of resistance using CLSI and Etest methods.^17,40,43^ A total of 13 studies reported flucytosine susceptibilities with resistance ranging from 0% to 11.6%. The single study reporting >10% flucytosine ‘resistance’ utilised epidemiological cutoff values as interpretive breakpoints for flucytosine are not provided by CLSI or EUCAST.^44^ Since non-wild-type rates do not equate to clinical resistance, these findings should be interpreted with caution.

### Preventability

This section addresses risk factors for *C. parapsilosis* infection and their mitigation. Three studies (with an unclear or high risk of bias) examined risk factors, but they did not undertake regression analyses to define magnitude.^12,13,19^ Risk factors described for *C. parapsilosis* infection were similar to those established for other forms of candidaemia, such as intensive care admission or intervention and the presence of a CVC. For *C. parapsilosis*, they included the prior use of echinocandin antifungal agents. Two studies^14,33^ discussed risk factors for all candidaemia without specifically addressing *C. parapsilosis*, and 12 reported on risk factors for mortality associated with *C. parapsilosis* bloodstream infection rather than the risk of infection *per se*. A multi-variate analysis in one study found that for patients with candidaemia with *C. parapsilosis sensu lato*, shock and abdominal surgery were independently associated with poorer outcomes, whilst antifungal therapy and central catheter removal were independently associated with better outcomes.^19^

Six studies assessed preventative measures. Several studies reported nosocomial outbreaks or transmissions of *C. parapsilosis* candidaemia via biofilm on prosthetic material or CVC but also potentially being carried on the hands of healthcare workers,^3,8,16,20,33,37,41,45,46^ which reflect that effective surveillance and infection-prevention programmes can be preventive measures for *C. parapsilosis* fungemia, especially CVC-related candidaemia. A study from Spain reported the success of an intervention (in which staff were trained on hospital catheter-related blood-stream infection reduction guidelines) and (undefined) care bundles were implemented. In adult patients, the incidence of catheter-related *C. albicans* and *C. parapsilosis* infections fell from 0.53 to 0.36 cases/1000 admissions/year.^47^ Although not related to infection prevention, three studies reported that early CVC removal (≤48 h) reduced the mortality rate.^9,10,30^ However, one study showed no difference in mortality with early CVC removal.^48^

### Annual incidence

Table 9 outlines the nine studies that reported incidence rates of candidaemia due to *C. parapsilosis*, although the denominator in these is generally institutional rather than the general population. One study, with a low risk of bias, reported a population incidence rate, making a crude estimate of 2.10 per 100 000 population per year.^10^

**Table 9.** Annual incidence.

Author, year	Study type	Study design	Study period	Country	Population description	Number of patients	Number of cases/Number of population
Barchiesi, 2016	Retrospective cohort study	Single- centre	January 2010–December 2014	Italy	Patients with candidaemia	270 (63 *C. parapsilosis* complex)	0.4 episodes/1000 hospital admissions
Chen, 2015	Retrospective cohort study	Single- centre	2000–2012	Taiwan	Adults >18	323	0.16/10 000 patient days in 2000 0.48/10 000 patient days in 2003
Escribano, 2018	Retrospective cohort study	Single- centre	January 2007–December 2014 (in 2 periods: 2007–2010 (period 1) and 2011–2014 (period 2)	Spain	Adults, paediatric and neonatal	432 in total *Candida parapsilosis* (*N* = 156) *Candida albicans* (*N* = 276)	Overall candidaemia 1.49/1000 admissions/year (mean) Overall mean 0.43/1000 admissions/year for *C. parapsilosis* (0.47 period 1 vs. 0.39 period 2)
Fernandez-Ruiz, 2014	Other: sub-analysis of completed prospective, multi-centre, population-based surveillance programme	Multi-centre	May 2010–April 2011	Spain	Mixed adult, paediatric and neonatal	773 episodes in >190 patients (*C. parapsilosis sensu stricto* only)	2.10/100 000 population/year
Lin, 2015	Retrospective cohort study	Single- centre	June 2008–June 2012	Taiwan	Mixed adult and paediatric	77	0.01–0.04/1000 inpatient days
Medeiros, 2019	Retrospective cohort study	Single- centre	January 2011–December 2016	Brazil	Adult (+1 child)	68	2.23 cases/100 admission—all candidaemia
Pinhati, 2016	Cross sectional study	Single- centre	July 2011–February 2012	Brazil	Adults ≥18	40	6/1000 patients—day admissions (all candidaemia)
Sun, 2019	Retrospective cohort study	Single- centre	1 March 2012–28 February 2018	China	Adults and paediatric	323	1.3 episodes/1000 hospital admissions
Van Schalkwyk, 2019	Lab surveillance study	Multi-centre	2016–2017	South Africa	Adults and paediatric	2600	32.98/100 000 admissions

**Table 10.** Proportion of *C. parapsilosis* causing candida infection.

Author, year	Study type	Study design	Study period	Country	Level of care	Population description	Number of patients	Prevalence (%)
Aboutalebian, 2019	Cross sectional study	Single-centre	January 2016–January 2017	Iran	Tertiary	Patients with otomycosis	97	22/92 (23.9%) *C. parapsilosis* was the most common species
Barchiesi, 2016	Retrospective cohort study	Single-centre	January 2010–December 2014	Italy	Tertiary	Patients with candidaemia	270 (63 *C. parapsilosis* complex)	63/270 *C. parapsilosis* complex amongst candidaemia (23%)
Caggiano, 2017	Retrospective cohort study	Single-centre	January 2007–December 2015	Italy	Tertiary	NICU patients with candidaemia	41	*Candida parapsilosis sensu stricto* was isolated with the highest frequency (58.5%)
Chapman, 2016	Prospective cohort study	Multi-centre	August 2014–July 2015	Australia	Tertiary	Episodes of candidaemia	526 patients with confirmed species for 498 *Candida spp*. (82 *C. parapsilosis* complex)	82/498 (16.5%) *C. parapsilosis* complex
Pharkjaksu, 2018	Laboratory epidemiology and sensitivity study	Single-centre	January 2011–December 2015	Thailand	Tertiary	Not stated	96	Average prevalence 13.99% *C. parapsilosis* complex for invasive candidiasis (68.7% *C. parapsilosis sensu stricto*)
Wang, 2016	Laboratory surveillance study	Single-centre	2010–2014	China	Tertiary	Not stated	155	*Candida parapsilosis*/all yeast infections Total 212/443 (48%) 2010: 9/68 (13%) 2011: 20/74 (27%) 2012: 11/58 (19%) 2013: 118/150 (79%) 2014: 54/93 (58%)
Yamin, 2020	Cross sectional retrospective study	Single-centre	1 January 2001–31 December 2018	Malaysia	Tertiary	All in patients—adult and paediatric	1175	343/1175 (29.2%) of bloodstream isolates
Zhang, 2020	Retrospective cohort study	Single-centre	January 2012–December 2018	China	Tertiary	Adult >14 years old	232	43% of study sample of 172 surgical patients

**Table 11.** Trends in the last 10 years.

Author, year	Study type	Study design	Study period	Country	Number of cases/number of population	Population type	Prevalence	Pattern
Asadzadeh, 2017	Retrospective cohort study	Multi-centre	01/2012–12/2015	Kuwait	ND	ND	ND	Increasing fluconazole resistance
Boan, 2019	Retrospective cohort study	Multi-centre	01/2005–12/2014	Australia	ND	ND	ND	Prevalence of *C. parapsilosis* complex increased from 13.1% to 16.7% (*P* = .670)
Chen, 2015	Retrospective cohort study	Single-centre	2000–2012	Taiwan	0.16/10 000 patient days 2000 0.48/10 000 patient days 2003	ND	ND	Minor decrease incidence - otherwise relatively unchanged
Lin, 2015	Retrospective cohort study	Single-centre	June 2008–June 2012	Taiwan	0.01–0.04/1000 inpatient days	All	ND	Mild decrease not statistically significant
Mesini, 2020	Other: prospective cohort with retrospective cohort inclusion	Single-centre	2008–2011 2012–2016	Italy	All candidaemia 1.97 episodes/10 000 patient days (2008) 4.59/10 000 patient days (2016)	All	ND	Increasing incidence IRR (incidence rate ratio): 1.04, *P* < .001 for *C. parapsilosis*
Wang, 2016	Other: laboratory surveillance study	Single-centre	2010–2014	China	ND	ND	2010: 9/68 (13.2%) *C. parapsilosis* 2011: 20/74 (27%) 2012: 11/58 (19%) 2013: 118/150 (78.7%) 2014: 54/93 (58.1%)	Increasing
Yamin, 2020	Other: cross sectional retrospective study	Single-centre	1 January 2001–31 December 2018	Malaysia	ND	ND	29.2% (343/1175) of bloodstream isolates	Shift from *C. albicans* predominance (2001) to *C. parapsilosis* for most following years

Note: ND, not determined.

### Distribution

Many studies reported the proportion of *C. parapsilosis* of all *Candida* bloodstream isolates ranging from 10% to 43%.^8,14,20,21,24–33^ The proportion was as high as 58% (Brazil)^49^ and 70% (Italy),^8^ reported in association with outbreaks of *C. parapsilosis* infection in ICU settings. A study from Iran reported the leading cause of otomycosis cases to be *C. parapsilosis* (23.9%).^50^

### Current global distribution

*Candida parapsilosis* is globally distributed, as evidenced by studies included in this review from Europe, America, Africa, Oceania, and Asia. However, data were limited in many low- and middle-income countries.

### Trends in last 10 years

Seven studies reported on the observed trends for *C. parapsilosis*. Three studies (from China, Italy, and Australia) showed an increase in incidence or prevalence,^20,24,37^ and two (both from Taiwan) showed decreases in incidence.^19,48^ A Malaysian study^51^ reported on a change of predominant *Candida* species for candidaemia from *C. albicans* in 2001 to *C. parapsilosis* from 2002 to 2018. No studies provided trend data across the whole study period.

## Discussion

The findings of this systematic review underscore the increasing public health concern posed by *C. parapsilosis* infections. The proportion of candidaemia cases caused by *C. parapsilosis* increased and ranged from 10% to 43%, making this pathogen an important focus for future research. The 30-day crude mortality rates in patients with *C. parapsilosis* bloodstream infections ranged from 17.5% to 46.8% across studies. This is comparable to the mortality rates of *C. albicans* and is unacceptably high. However, no studies reported attributable mortality, which might reflect gaps in surveillance and the quality of available data.

No studies reported detailed health economic data in terms of hospital costs. However, most studies reported an extended length of hospital stay, ranging from 2 to 12 weeks. It was impossible to ascertain whether the prolonged length of hospital admission was directly attributable to infection due to data limitation. Given that several guidelines recommend a minimum of 2 weeks of therapy for candidaemia and longer for invasive infections with complications, it stands to reason that most patients will receive at least two weeks of inpatient care.

Whilst no captured studies reported on specific complications or sequelae for invasive infection due to *C. parapsilosis*, based on complications for other *Candida* species and considering the populations affected, it is reasonable to assume that complications could include neonatal meningoencephalitis, which may result in prolonged admissions and/or neurodevelopmental delay. Other secondary-seeded infections, such as infective endocarditis, endophthalmitis, and vertebral osteomyelitis, are also plausible and would likely result in long-term morbidity consequences. Given that case reports were excluded from this systematic review, complications that are likely to be rare could be missed, and more explicit estimates of the non-fatal burden are urgently needed.

Multiple centres from various countries reported fluconazole resistance in *C. parapsilosis* ranging from 1.9% to 8.8%.^10,14,24,25,27,33–35,38,40,48,52^ However, much higher rates of resistance to fluconazole have been reported in specific geographic regions outside the study period, such as surveillance from Southern Africa in 2009–2010, which reported fluconazole resistance rates between 35% and 54%^53–55^ perhaps attributed to clonal transmission of fluconazole resistance isolates. It was also noted that fluconazole-resistant isolates had significant cross-resistance to voriconazole.^53^ These rates of resistance to azoles are of concern, though fortunately, resistance to echinocandins and amphotericin B was rarely reported, although it is noted that *C. parapsilosis* is intrinsically less susceptible to echinocandins than other *Candida* species. Studies that specifically addressed biofilm mass and antifungal susceptibility demonstrated that susceptibility to all antifungal agents within biofilm was much reduced—this may have significant clinical implications given the association of *C. parapsilosis* with indwelling medical devices.

Whilst many studies reported on nosocomial outbreaks or transmission of *C. parapsilosis*, there was a lack of solid evidence for effective prevention methods. Early removal of central lines reduces the incidence of infection, suggesting that infection-prevention programmes in the hospital that include CVC management in a care bundle may be effective as a preventative strategy. This is an area that requires more studies to generate policy-informing evidence.

The studies in this systematic review show that *C. parapsilosis* is an emerging fungal pathogen with a wide global distribution. However, data from some WHO regions were missing from this review. The lack of comprehensive data from low-income settings raises concerns. Efforts to enhance international collaboration, data sharing, and surveillance are crucial to better understand regional variations and to inform targeted interventions. Moreover, the scarcity of data on morbidity impacts and complications associated with *C. parapsilosis* infections warrants further investigation, given the potential for long-term sequelae. Sustainable systematic global surveillance is required to ensure that critical regional variations in incidence and epidemiology can be monitored for this as well as other fungal pathogens.

## Conclusion

This systematic review has identified several critical knowledge gaps. There is a significant lack of data from low-income settings, especially related to incidence, prevalence among candidaemia cases, mortality, and non-fatal burden. Most reports on susceptibility came from laboratory surveillance without clinical correlation. This makes it difficult to assess the impact of observed changes in susceptibility and is a barrier to developing clinical breakpoints.

There was a general lack of clinical data, especially around morbidity impacts. We have summarised current data on *C. parapsilosis* and highlighted where ongoing research and surveillance are required to better understand this increasingly important pathogen. Specifically, research should focus on large clinical cohorts, which can be used to better understand the burden of disease and form the basis of future prevention and therapeutic studies. Health economic impacts must be included. It will also be important to enrol both adult and paediatric patients across different patient cohorts and harmonise outcome measures to allow comparisons across time and geographies.

In conclusion, this systematic review provides valuable insights into the clinical and public health implications of *C. parapsilosis* infections. The identified knowledge gaps underscore the need for concerted efforts in research, surveillance, and prevention. Addressing the gaps in our understanding of this pathogen’s impact on patient outcomes and healthcare systems is essential to guide intervention strategies and improve patient care and outcomes.
